# Redefining HIV care: a path toward sustainability post-UNAIDS 95-95-95 targets

**DOI:** 10.3389/fpubh.2023.1273720

**Published:** 2023-10-19

**Authors:** Godfrey Musuka, Enos Moyo, Diego Cuadros, Helena Herrera, Tafadzwa Dzinamarira

**Affiliations:** ^1^International Initiative for Impact Evaluation, Harare, Zimbabwe; ^2^School of Nursing and Public Health, University of KwaZulu-Natal, Durban, South Africa; ^3^Digital Epidemiology Laboratory, Digital Futures, University of Cincinnati, Cincinnati, OH, United States; ^4^School of Pharmacy and Biomedical Sciences, Faculty of Health and Science, University of Portsmouth, Portsmouth, United Kingdom; ^5^School of Health Systems and Public Health, University of Pretoria, Pretoria, South Africa

**Keywords:** HIV programmes, UNAIDS 95-95-95, low and middle-income countries, transition, considerations

## 1. Introduction

In 2021, around 38 millionpeople were living with HIV (PLHIV) globally. In the same year, the number of new HIV infections worldwide was 1.5 million, while 650,000 died from AIDS-related illnesses (1). Low and middle-income nations (LMICs), especially those in sub-Saharan Africa (SSA), have been hardest hit by the HIV epidemic. Apart from the death toll, the epidemic severely impacted LMICs' health systems. The HIV epidemic led to a shortage of healthcare workers (HCWs) in LMICs due to death and absenteeism from work as a result of HIV-related illnesses, especially in the years before the introduction of antiretroviral treatment (ART) (2). Before ART, opportunistic diseases such as cryptococcal meningitis, diarrhoeal disorders, and tuberculosis significantly increased the demand for healthcare. As patients stayed in hospitals longer due to opportunistic infections, the need for hospital beds increased (3). The increased demand for healthcare also led to an increase in overall healthcare costs. In many countries, especially the LMICs, the health expenditure on HIV escalated, which had a knock-on effect, reducing spending on non-HIV diseases (4).

Over the past few decades, significant progress has been made in containing the HIV epidemic globally. With only 1.3 million new infections recorded worldwide in 2022, HIV incidence has decreased considerably. This is the lowest number of new conditions reported in decades (5). The number of AIDS-related deaths in 2022 was 69% lower than the number recorded at the peak in 2004. It was reported that 76% of PLHIV were on ART, and 71% were virally suppressed. Although much progress has been made, more must be done in many countries to achieve epidemic control (5). The Joint United Nations Program on HIV/AIDS (UNAIDS) recently revealed that Botswana, Eswatini, Rwanda, the United Republic of Tanzania, and Zimbabwe have already achieved the UNAIDS 95-95-95 targets (5). That means 95% of the PLHIV know their HIV status, 95% of the PLHIV are on lifesaving ART, and 95% of people on ART are virally suppressed (6). A further 16 other countries, eight of them in SSA, the region which accounts for 65% of PLHIV, are also close to reaching these targets (5). This is a major milestone, and it is essential to consider what steps should come next. This article discusses issues that need to be considered as countries transition into the post-UNAIDS 95-95-95 targets era.

## 2. Considerations

### 2.1. Integration of health services

One of the key challenges that countries that have achieved the 95-95-95 targets may face is how to integrate HIV programs into the rest of the health system out of the multiple ways in which these can be incorporated. A successful integration strategy should consider the specific needs and contexts of each setting. Some of the integration strategies include:

- *Co-located services*: This involves offering services for HIV and other chronic diseases in the same facility. This has been successfully implemented in some settings, with HIV care integrated into primary healthcare clinics or non-communicable disease (NCD) care provided in HIV clinics (7). In many countries in southern and eastern Africa, HIV services are provided using separate rooms/buildings within the clinic or hospital compounds.
- *Service integration*: This can involve a “one-stop-shop” model, where patients receive all their care, from HIV treatment to NCD management to mental health services, from the same provider or team (8). This will involve ensuring that clinical staff at HIV service points have adequate supplies, equipment and consumables to address non-HIV related health needs of clients such as NCDs when they present.
- *Integrated training and capacity building*: Healthcare workers should be trained to manage both HIV and other chronic diseases. This can involve cross-training staff, integrating HIV and NCD guidelines, and building capacity for mental health services (9). This integration can occur firstly at the pre-service training of health workers and for those already in service during periodic refresher courses.
- *Integrating data systems*: Existing data systems for HIV can be expanded to include data on other chronic diseases, allowing for better monitoring and management of patients' health (10). HIV-related Electronic health systems in countries with high HIV burden in line with funding priorities of funders such as PEPFAR tend to be in silos. There is a need going forward to ensure that the electronic health system is client-centered and not disease-specific so that it's possible to collect data in an integrated matter whenever a client visits a facility for whatever reason.

The integration strategies proffered above are broad and can be seen as step-wise approaches that countries can consider. Depending on the country's context some steps can happen before others or can be skipped altogether. Integration of services is essential because the increasing life expectancy (11) and the side effects of ART are leading to more co-morbidities among PLHIV (12). There is also a higher prevalence of mental health disorders among PLHIV than the general population (13). This shift necessitates re-evaluating how we deliver HIV care, moving from a disease-focused approach to a more patient-centered, integrated model that addresses the full spectrum of a patient's health needs. One systematic review and meta-analysis study revealed that integrated services had superior values for HIV care cascade outcomes such as uptake of HIV testing and counseling, ART coverage and initiation, time until ART initiation and viral suppression, and retention in HIV treatment. Additionally, utilization of non-HIV services and the success of non-HIV-related disease and condition treatments were higher in integrated services (14).

The joint delivery of services may optimize healthcare spending due to less duplication of procedures and the synergies that will be created. The allocation of resources and procedures may both benefit from integration. Existing resources and infrastructure, such as diagnostic equipment, laboratories, and health personnel, can be utilized for HIV and non-HIV-related services. This enhances resource efficiency and broadens the scope of services offered in a single visit. Moreover, integrating services contributes significantly to the continuity and quality of care, as individuals receive comprehensive care for all their health needs in one location (14). In addition, integrating HIV programs with other health services may benefit patients since it could reduce waiting times and the inconvenience of utilizing several health services separately (15). Furthermore, integration may reduce stigma and negative community attitudes toward PLHIV (16). Integration of HIV programs with other health services may help boost the sustainability of the global HIV response (17). This integration would benefit by being first implemented in selected pilot health facilities to generate lessons that can be used to increase the likelihood of more successful national roll-outs.

It should be understood, though, that service integration is not a quick fix for improving the quality and accessibility of care. Several challenges may be encountered and need to be taken into consideration when implementing integration of services (9). One of the challenges is an increase in healthcare costs arising from reduced specialization and the reduced efficiency gains it brings as a result of joint service delivery (14). Integration of services may also overburden healthcare providers who are already few and overworked, especially in low and middle-income countries (LMICs), which could lead to increased staff turnover (18). Another challenge that may be faced is that the healthcare workers (HCWs) would require appropriate training to provide these services (9). To address these challenges, we suggest that HCWs receive training tailored to these needs, supplemented by incentives aimed at motivating them to remain working in LMICs. Furthermore, we recommend the implementation of task shifting to reduce the workload of HCWs, where some tasks are removed from doctors, nurses, and pharmacists, and delegated to support staff such as nurse assistants and pharmacy assistants, after they have received adequate training.

For integration to succeed, we suggest that a population needs assessment is conducted before it is implemented (9). We also suggest that patients, communities, and HCWs should be engaged during both the planning and implementation phases of the integration to ensure their buy-in (7). We advocate for a strong political will and leadership by healthcare managers and recommend the formulation of guidelines, protocols, checklists, and algorithms for integration (19). This will help to ensure that PLHIV receives the same quality of care for all their health needs. We also suggest aligning service funding to ensure equitable funding distribution for the different services. We also recommend the implementation of organizational culture change that supports integration in healthcare institutions (9). The transition to integrated HIV/AIDS services will be complex and needs to be managed well. Instead of a sudden shift from vertical services to horizontal (full integration), health systems may need to find transit through a diagonal approach which is a mix of both vertical and horizontal provisionally (20).

Integration is a crucial strategy for achieving Universal Health Coverage (UHC), aiming to ensure everyone can access the health services they need without facing financial hardship. By making health systems more efficient and responsive to the needs of their populations, integrated services can significantly contribute to achieving this goal. However, careful planning and implementation are essential to improve health outcomes (10). This will require cross-sectoral coordination, capacity building of HCWs, strengthening of health systems, adequate financing, and meaningful involvement of communities and PLHIV (21). However, the potential benefits for patients and health systems make this a worthy strategy. As we move toward a future of sustained HIV care, integrating services to manage chronic disease comorbidities will be crucial in ensuring that PLHIV continues to receive the comprehensive care they need.

### 2.2. Targeted HIV prevention services

HIV prevention services that were previously cost-effective may no longer be so after achieving the UNAIDS 95-95-95 targets because of the smaller number of people still at risk of HIV infection. We, therefore, suggest that policymakers tailor prevention services to those most at risk of HIV infection, such as key populations, for them to be cost-effective. Furthermore, we recommend that extensive population-based HIV surveys be replaced with surveys targeting only populations at high risk of HIV infection since this will require fewer resources while providing useful data.

In countries where HIV prevalence would have dropped to <5%, we suggest that HIV testing algorithms be changed from the double assay testing strategy used by countries with more than 5% HIV prevalence to the triple assay testing strategy (22). We also recommend the promotion of long-acting and extended delivery drugs such as Dapivirine vaginal ring (DVR) and long-acting cabotegravir (CAB-LA) injection for HIV pre-exposure prophylaxis (PrEP) as they are not affected by adherence issues like oral PrEP (23).

### 2.3. Differentiated service delivery models

The World Health Organization (WHO) advised differentiated service delivery (DSM) models for HIV services in 2015, particularly in countries with high incidence rates. The WHO stressed the need to improve service quality and access, adherence and retention, clinical outcomes, efficacy, and the cost of the services, in addition to strengthening the continuum of HIV care (24). We recommend LMICs leverage the lessons learned in DSD models for HIV to expand their use in integrated services. We suggest that out-of-facility individual models such as community pharmacies, outreach models, and home deliveries be promoted in the provision of integrated services since they reduce traveling expenses for patients and the work burden of the HCWs. DSD models may also improve adherence and retention in care. The reduction in the burden of work for HCWs may result in an improvement in the quality of care at the healthcare facilities (25). However, for such models to be effective, the targeted population groups must be involved in their design and implementation. We also recommend providing information on DSD models to the targeted population groups (26).

### 2.4. Funding options for sustainability

To determine which nations are eligible for funding, the Global Fund considers both indices of disease burden and the gross national income per capita of each country. As countries achieve HIV pandemic control, funding from the Global Fund and other international organizations is anticipated to decline (27). Development assistance for HIV and AIDS, which reached its peak in 2012 at US$12.0 billion, had fallen by a quarter by 2017, partly due to global economic challenges and partly due to the reduction in the disease burden (28). Inadequately planned transitions away from donor funding may present significant challenges to sustaining the progress made in population health for LMICs since they may fail to maintain the necessary investment required for domestic health financing (27). As a result, we recommend that LMICs find innovative strategies to fund their HIV programs and other health services. One of the strategies we recommend is increased government spending on health by LMICs. Although African countries agreed to allocate at least 15% of their government budget to the health sector in the Abuja Declaration of 2001, which was later reaffirmed in the Maputo Declaration of 2003, only a few countries have met this target due to competing national priorities (29). LMICs' governments should, therefore, be encouraged to spend more on health in the face of diminishing donor funding. We also recommend the local pooling of resources for healthcare funding. This may require approaching the business community to help fund some programs while other programs are funded by private-public partnerships, and charging excise duty on some goods (30).

## 3. Conclusion

LMICs, especially those in SSA, have been hardest hit by the HIV epidemic. Apart from the death toll, the epidemic severely impacted LMICs' health systems. Over the past few decades, significant progress has been made in containing the HIV epidemic globally. Several countries have already achieved the UNAIDS 95-95-95 targets, and others are close to achieving these targets. Countries that have earned the UNAIDS 95-95-95 targets should start planning how to transition to a post-UNAIDS 95-95-95 targets era. Although several issues need to be considered, one crucial issue that should be addressed is integrating HIV services into other health services. This is because the increasing life expectancy of PLHIV and the side effects of ART are leading to more co-morbidities among them. Integration will bring better HIV care cascade outcomes and non-HIV-related disease outcomes. Barriers to integration should, however, be addressed for it to succeed.

## Author contributions

GM: Conceptualization, Writing—original draft. EM: Writing—review and editing. DC: Writing—review and editing. HH: Writing—review and editing. TD: Conceptualization, Writing—original draft.
